# Factors effecting recurrence and progression of high grade non invasive bladder cancer treated by intravesical BCG

**DOI:** 10.12669/pjms.302.4117

**Published:** 2014

**Authors:** Muhammad Kashif Khan, Irfan Ahmed, Syed Johar Raza

**Affiliations:** 1 Muhammad Kashif Khan, FCPS, Shaukat Khanum Memorial Cancer Hospital & Research Center Lahore, Lahore – Pakistan.; 2 Irfan Ahmed, FRCS, Shaukat Khanum Memorial Cancer Hospital & Research Center Lahore, Lahore – Pakistan.; 3 Syed Johar Raza, FCPS, Shaukat Khanum Memorial Cancer Hospital & Research Center Lahore, Lahore – Pakistan.

**Keywords:** Non-muscle invasive bladder cancer, High grade, BCG, Recurrence, Progression

## Abstract

***Objective: ***The rate of recurrence in high grade non muscle invasive bladder cancer (NMIBC) is 70% with progression rate of 15-40% at 5 years. The treatment of high grade NMIBC is intravesical BCG therapy, however for high risk cases radical cystectomy is recommended. In this study we determined the response of high grade NMIBC to BCG therapy and the factors affecting it in south Asian population.

***Methods: ***This retrospective cohort study was conducted on 64 patients treated with intravesical BCG for high grade NMIBC from Dec 2008 to July 2012. Smoking, tumor size, location and multiplicity were taken as prognostic factors. Recurrence and progression were determined by cystoscopy and upper tract imaging according to European Association of Urology guidelines. The association of prognostic factors with recurrence and progression was determined.

***Results: ***The rate of recurrence and progression was found to 45.8% and 27.1% respectively after a mean follow up 28.36 months. Smokers had 4 times greater odds of progression of tumor as compared to non-smokers. Patients with large tumors had 6.7 times greater odds of progression as compared to patients with small tumors.

***Conclusion: ***Smokers with large and multiple high grade NMIBC constitute the high risk group. These patients may be offered early radical cystectomy and advised to stop smoking.

## INTRODUCTION

Carcinoma of the bladder is an important healthcare problem globally. The worldwide age standardized incidence rate (ASR) is 10.1 per 100,000 for males and 2.5 per 100,000 for females.^1^ Bladder cancer is the 4^th^ most common malignant tumor among males accounting for 5.6% of all reported cancers in Pakistan with an ASR of 8.9 per 100,000 individuals.^2^

Transitional cell carcinoma (TCC) accounts for more than 90% of all the bladder cancers out of which 70%are non-muscle invasive bladder cancer (NMIBC) at diagnosis.^3^ High grade NMIBC has a recurrence rate of 70% with 15-40% risk of progression as compared to less than 5% for low grade tumors at 5 years. Progression to muscle invasive disease increases to almost 50% in recurrent high grade tumors at 5 years.^4^^,^^5^ The mainstay of treatment for high grade NMIBC is intravesical Bacille Calmette-Guerin (BCG) therapy after transurethral resection, however in selected cases and BCG non-responders radical cystectomy is recommended.^6^ Other intra-vesical agents are also being used but none has surpassed the efficacy of BCG.^7^^,^^8^ It acts by inducing cytokine and direct cell to cell cytotoxicity response which targets tumor cells. Disease free survival at 5 years after radical cystectomy is 90% when carried out for NMIBC as compared to only 30-50% for muscle invasive disease.^9^^,^^10^ It is important to understand the factors that could predict BCG failures and help identify patients for early radical cystectomy.^11^

Majority of the large international trials conducted for determination of recurrence and progression of NMIBC used the 1973 WHO grading system. This grading system is being phased out and replaced by the 2004 WHO grading system, questioning the applicability of these results to the current histological grading.^12^^,^^13^ There is paucity of data on NMIBC from Pakistan, providing a poor insight regarding the recurrence and progression following BCG therapy. This lack of data consequently makes radical cystectomy, which is a major undertaking a rather difficult decision in our population with NMIBC. In this study we determined the response of NMIBC to intravesical BCG therapy and identified the factors which could affect recurrence and progression in south Asian population.

## METHODS

This retrospective cohort study was conducted on patients with high grade non muscle invasive bladder cancer (n=64) managed by intravesical BCG therapy at the department of surgical oncology Shaukat Khanum Memorial Cancer Hospital and Research centre Lahore, Pakistan from Dec 2008 to July 2012. All the tumors were resected at our setup to establish the diagnosis. Those patients who did not complete six cycles of intravesical BCG nor had a follow up of six months were excluded from the study. The list of patients was retrieved from a database of patients with bladder cancer which is prospectively maintained and the records were reviewed by the surgical oncology fellow.

The study was approved by the Institutional Review Board (IRB). Data collection included socio-demographic, disease and outcome related variables. The history of smoking (more than 1 pack year) and tumor characteristics on initial cystoscopy were taken as prognostic markers. Tumor size more than 3cm, multiplicity and location were noted. Mitomycin-C 40 mg was instilled intra-vesically after transurethral resection of bladder tumor (TURBT). Stage (2002 TNM Classification) and grade (as per WHO/ISUP 2004) of the resected specimens were established by a dedicated uropathologist.^13^^,^^14^ All the patients with high grade NMIBC were included in the study. They were treated with intravesical BCG therapy which was given once weekly for six weeks. Similar strain and dose oftice BCG (500 miu of Mycobacterium bovis diluted in 50 ml of normal saline) was used for all patients using a standard instillation technique retaining inside the bladder for an hour. Those patients who had recurrence were administered maintenance therapy.

Surveillance was carried out with cystoscopic examination and upper tract imaging in concordance with the European Association of Urology guidelines.^6^ Tumors found at first check cystoscopy and on subsequent examinations were resected and sent for histopathological examination. Recurrence was defined as tumors with same stage; while progression was defined when the tumor involved the detrusor muscle, had nodal or distant metastasis.

Statistical analysis was conducted using Statistical Package for Social Sciences (SPSS) version 19. Descriptive analysis was done for age, time to recurrence, time to progression and follow up in months. Frequency and percentages were calculated for gender, smoking, recurrence, progression, tumor size, location and multiplicity. Chi-square test was used to determine significance of prognostic markers. A p-value of less than 0.05 was considered as significant. Keeping in view the small sample size, multivariable logistic regression analysis with forward progression was done to determine the association of prognostic factors with recurrence and progression.

## RESULTS

A total of 64 patients underwent intravesical BCG therapy for high grade NMIBC. Five patients either could not complete the therapy or lost to follow up and were excluded from the study. Patient and tumor characteristics and the response to therapy are given in Table-I. On determination of significance of each variable for recurrence and progression; we identified smoking to be significant for progression only while multiplicity of tumors had significant effect on recurrence only. Large size of tumor was statistically significant variable associated with both recurrence and progression (Table-II). On multivariate analysis with forward progression, the patients with multiple tumors were found to have 3.8 times greater odds of recurrence as compared to patients with single lesion. Smokers had 4 times greater odds of progression of tumor as compared to non-smokers. Patients with large tumors had 6.7 times greater odds of progression as compared to patients with small tumors (Table-III).

## DISCUSSION

Intravesical BCG therapy is being used for last 30 years to treat NMIBC after transurethral resection. Its role in reducing the recurrence rate to half and effect on progression is well established. European Organization for Research and Treatment of Cancer (EORTC) has developed a scoring system which predicts the recurrence and progression rates at 1 and 5 years. Its inclusion period was 1979-1989 when patients were undertreated in comparison with the current guidelines, and only 171 out 2596 of the patients were treated with intravesical BCG.^15^^,^^16^ Fernandez-Gomez J et al published data of three randomized studies conducted by the Spanish Urological Club for Oncological Treatment (CUETO) from 1990 to 1999 on NMIBC. In this largest multivariate prognostic factor analysis to date, on patients treated by BCG, there were 235 patients with high grade NMIBC. They determined that multiplicity, female gender and TIS are predictors of recurrence whereas predictors for progression included age, high grade, T1 stage and recurrence at first check cystoscopy. They also made CUETO scoring model which is more helpful in patients being treated by intravesical BCG therapy as compared to EORTC risk table.^17^ These scoring systems are being used in clinical practice but their main limitation is that they use 1973 WHO histological grading of bladder cancers. Interestingly these studies did not take into account the impact of smoking. The new research is being done using 2004 WHO grading as it has high intra observer reproducibility, and the new grading system requires validation in more clinical trials. Our study evaluates the impact of predictors on high risk NMIBC patients in particular, with response to intravesical BCG therapy. In addition there is ongoing research at molecular level to determine response of NMIBC to BCG therapy and in future it is likely that validated molecular markers will be added to the risk tables.^18^

**Table-I:** Description of patient and tumor characteristics and response to therapy

*Patient Characteristics*	
Age, years Mean±SD (range)	59.86 ± 9.85 (38-78)
Male gender, n (%)	53 (89.8)
Follow up, months Mean ± SD	28.36 ± 10.64
Smokers, n (%)	32 (54.2)
*Tumor Characteristics*	
Large size, n (%) range	31 (52.5)1-10cm
Neck/Trigone location, n (%)	27 (45.8)
Multiple, n (%) range	29 (49.2)1-8
*Response to therapy*	
Time to recurrence, months Mean ±SD	6.77 ± 6.78
Time to progression, months Mean ±SD	10.1 ± 6.91
First check cystoscopy, n (%) Recurrence Progression	12 (20.3) 4 (6.8)
Overall recurrence, n (%)	27 (45.8)
Overall progression, n (%)	16 (27.1)

**Table-II:** Chi-Square test to determine significance of each variable

*Variable*	*Outcome*					
	*Recurrence*		*P value*	*Progression*		*P value*
	*No (n=32)*	*Yes (n=27)*		*No (n=43)*	*Yes (n=16)*	
Smoking	15	17	0.21	20	12	0.05
Multiplicity	11	18	0.01	18	11	0.06
Neck/trigone	12	15	0.16	20	7	0.85
Large size	13	18	0.04	18	13	0.007

**Table-III:** Multivariate logistic regression with forward progression analysis to evaluate variable association with recurrence and progression

*Recurrence*		
Variable	Odds ratio(95% C.I)	P value
Multiplicity	3.81 (1.29-11.27)	0.015
*Progression*		
Smoking	4.02 (1.01-15.88)	0.04
Large size	6.76 (1.58-28.85)	0.01

Jancke G et al concluded in their study that tumors larger than 3 cm were associated with increased risk of recurrence but there was no impact of tumor size on progression. In our study we found that tumor size had a significant effect on both recurrence and progression.^19^ Vukomanovic I et al. showed that tumors involving bladder neck had poor response to BCG therapy and were more likely to recur.^20^ The largest single institution study with the longest follow up was reported by Palou J et al.^21^ They reported an overall recurrence and progression rates of 44% and 17%, respectively in 140 patients over a median follow up of 8 years. Their study concluded that female gender and involvement of bladder neck/prostatic urethra are associated with increased risk of recurrence and progression. With a similar recurrence but a higher progression rate, we were unable to establish any relationship between tumor location and response to treatment.^20^ Rianne JM et al. reported smoking as an independent risk factor for recurrence and progression.^22^ Their finding is consistent with our conclusion that smokers have four times greater odds of progression as compared to non-smokers. This shows that smoking is not only causative agent in bladder cancer but also plays a major role in disease progression.

In Pakistan scanty work has been done on NMIBC. Ather MH et al. reported a series of 92 patients with NMIBC out of which 8 patients had a high grade disease and had a follow up period of 12 months. The recurrence rate was 37% and there was significant concordance with EORTC’s predictive scores. The progression rate was 2.2% which is less than predicted due to small number of patients with high grade disease.^23^ In another study published by Mansoor et al, the rate of recurrence and progression was 68.4% and 14% respectively in 92 patients. They did not take into account the impact of prognostic factors and used the 1973 WHO grading. There were 17 patients with TaG3 and 14 patients with T1G3 disease.^24^ To our knowledge none of the studies have reported any factors that impact the recurrence and progression rates following BCG therapy.

 Our study reports its findings from a retrospective review of the database. This remains to be the most important limitation of this study. Various biases including cystocopic findings and preoperative parameters can be avoided when data is collected retrospectively. We have not considered the use of single instillation of therapeutic agent Mitomycin-C as one of the variants, as it is known to affect the recurrence rates.^25^ In recognition of the fact that bladder cancer can be significant disease burden for Pakistani population, we recommend development of a multi-institutional prospective study to establish a better level of evidence in the understanding of the NMIBC.

## CONCLUSION

Smokers with large and multiple high grade NMIBC are at higher risk of recurrence and progression despite intravesical BCG therapy.

## Authors Contribution:

**MKK: **Data Collection, analysis and script of paper.

**IA:** Idea, script of paper.

**SJR: **Data analysis and script.

## Notes

***Financial support:*** No funding or other financial support was received.
